# COVID-19’s Influence on the Future of Agile

**DOI:** 10.1007/978-3-030-58858-8_32

**Published:** 2020-08-18

**Authors:** Dennis Mancl, Steven D. Fraser

**Affiliations:** 6 IT University of Copenhagen, Copenhagen, Denmark; 7 University of British Columbia, Vancouver, BC Canada; 8 MSWX Software Experts, Bridgewater, NJ 08807 USA; 9 Innoxec, Santa Clara, CA USA

**Keywords:** Agile, COVID-19, Digital transformation, Virtual collaboration

## Abstract

As a result of the global COVID-19 pandemic, the way the world works, collaborates, and plays has changed. Commerce has stalled with travel, hospitality, education, retail, and health sectors particularly affected. This paper is based on an XP 2020 panel organized by Steven Fraser and featuring Aino Corry, Steve McConnell, and Rachel Reinitz. The panel discussed the impact of COVID-19 on knowledge workers, the acceleration of digital workplace transformation, and anticipated long term effects from the pandemic in the context of agile practices. Four key observations emerged from the discussion: First, virtual collaboration between those working from home is enabled by a variety of communication tools – substituting for face-to-face interactions. Second, agile work practices are harder to perform given the virtual nature of meetings and interactions. Third, communication tools are not always adequate for high-bandwidth or informal interactions, such as brainstorming, side discussions, or hallway conversations. Fourth, forming new teams and onboarding staff is challenging in a virtual work environment.

## Setting the Context: COVID-19’s Impact on Agile

In March 2020, the world changed due to the pandemic, which necessitated quarantines that impacted most if not all individuals, communities, and countries around the world. The pandemic had an almost immediate effect on the software community by limiting face-to-face collaborations and meetings.

Other consequences of the pandemic included supply chain and business continuity interruptions. The delivery of goods and services were affected by transportation challenges, including border closures, quarantines, and the need to prioritize medical supplies. COVID-19 has impacted many sectors of the global economy, including hospitality (restaurants, hotels, cruises, casinos, theme parks, etc.), travel (airlines, trains, buses, etc.), education (school and university), retail, and health. All of these sectors have struggled to adapt to a world where most people-to-people interactions are virtual.

Additionally, we now have virtual rather than face-to-face conferences. Technical interactions catalyzed by internationally recognized conferences such as ACM/IEEE’s ICSE and the Agile Alliance’s XP conference have been transformed to virtual experiences. Without face-to-face presence, the opportunity for interesting personal hallway conversations, which have long been a hallmark of such international exchanges, is lost. It is likely that virtual experiences will be *de rigueur* for the foreseeable future. The widespread adoption of work-from-home environments has accelerated the digital workplace transformation. A serendipitous consequence includes issues related to workforce compensation (based on location) and the move to widespread virtual interaction channels between work teams and with customers. In some ways, this move to mostly remote staff accelerates the possibility of offshoring and outsourcing, since if geography is removed as a limiting constraint, team members need not be co-located. Previously mandated face-to-face interactions have transformed to digital interactions through necessity – and knowledge workers are enjoying the benefits of reduced commute time while shifting employer expenses (e.g. real estate, heat, light, power, IT infrastructure) to personal home “overhead” costs.

The panel session began with an online poll of conference participants. The audience members were asked if they attended XP 2020 only because it was an online conference. Of the 80 conference attendees who responded, 30 indicated they had planned to attend the in-person conference, in contrast to 50 who indicated their attendance was enabled by the virtual nature of the conference. A similar response was elicited regarding plans to attend XP 2021: 47 participants said they would attend XP 2021 if it were virtual, in contrast to 31 who indicated that they would attend an in-person conference. This result suggests that conference organizers of the future should consider hybrid virtual-physical conferences to increase conference geographic reach even if COVID-19 is no longer a factor.

The three XP 2020 panelists, Aino Corry, Steve McConnell, and Rachel Reinitz, expressed their personal views on the world of virtual work in a discussion facilitated by Steven Fraser. Aino Vonge Corry is an agile software expert, a teacher, technical conference editor, and retrospectives facilitator working for her own consultancy company, MetaDeveloper. Corry is the author of a forthcoming book, *Retrospectives Antipatterns*, that is planned for release in fall 2020 [1]. Steve McConnell is CEO and Chief Software Engineer at Construx, a worldwide software consulting and training company. McConnell is also the author of *Code Complete* [2], the classic book on software development practices, as well as the recent *More Effective Agile* [3], a roadmap for software leaders. Rachel Reinitz is an IBM Fellow, and the CTO and Founder of the IBM Garage, an organization that consults with clients to define, build, and deploy cloud applications.

The panel impresario and co-author of this paper, Steven Fraser (Innoxec), advises on open innovation strategies to accelerate the development and adoption of technologies based on his work at HP, Cisco, Qualcomm, Nortel, and the Software Engineering Institute (SEI) at Carnegie Mellon University. Dennis Mancl, panel recorder and co-author of this paper, is an independent consultant on software technology and practices. He worked for many years for AT&T, Lucent, and Alcatel-Lucent. In his role as an internal software technology expert, he supported the ongoing education of developers in many technologies.

There were four main conclusions from the panelists. First, COVID-19 has made drastic changes in the way we do our daily work – it has affected our work schedule, our collaborations and travel, and we are still working to readjust our work-life balance. Second, agile work practices are harder to perform since casual conversations are limited due to the online nature of meetings and interactions. Third, although virtual collaboration tools for video chat and online meetings have improved since the turn of the century, current communications tools are still not as good as face-to-face for performing high-bandwidth and informal interactions, such as brainstorming, whiteboarding, side discussions, and hallway conversations. Finally, the process of forming new teams and onboarding new employees is challenging in a virtual work environment.

## COVID-19 Impact on Daily Work

Early in the panel discussion, McConnell presented a few results from a recent Construx study based on a survey of his clients’ recent experiences with a work-from-home environment [4]. McConnell noted that for most people, routine communications continue to work well in the new all-virtual environment, and some people feel more productive because they have fewer distractions. High-performing teams continued to do well in a virtual environment, however if a team suffered from interpersonal friction prior to COVID-19, the friction was exacerbated by working from home. McConnell further explained that the survey suggested that virtual collaborators felt discussions were more to the point, attendees weren’t distracted by side conversations, and meetings started on time and ran more efficiently.

Reinitz observed that the organization of work activities needed to be modified in the new virtual regime. She observed that one needs to resist merely “taking what you do face-to-face and now doing it virtually.” For example, her team used to run multi-day face-to-face workshops with clients. But in the new virtual COVID-19 environment, they made one important change to their process by spreading their workshops over additional days – scheduling a series of half-day sessions. Reinitz explained that a multi-day schedule made it easier for team members to schedule workshops since schedules were more flexible and not tightly constrained by travel logistics. Participants also had time for daily mini-retrospectives: “When we do workshopping, we usually do them in the morning – then in the afternoon, the team reflects and discusses what’s working.”

Corry added to Reinitz’s points, agreeing that we need to adjust the way we run some of our work activities. She observed that it is essential to discover what can be done in virtual meetings that would provide added benefits over being physically together. For example, Corry has frequently used “round robin” in virtual meetings – where each participant gets to speak in turn. It is more socially acceptable to use a round robin when virtual than in a face-to-face setting.

The panel discussion turned to speculation about back-to-work protocols when the dangers of COVID-19 diminish. Corry explained that many offices in Denmark had reopened in May and June, but that “some people thrived so much on working from home” that they would prefer to remain virtual.

McConnell echoed this observation. “I agree with Corry that we are seeing people who really don’t want to go back to work from the office. I see that in my own company. Some of that is about avoiding a potentially risky work environment, and some of it is just a work practice preference. I think right now it is impossible to separate which is which.” McConnell noted many tech workers have worked virtually (from home) for years, but the pandemic increased the use of online collaboration tools by less technical business partners. The increased familiarity with online collaboration likely will increase future acceptance for virtual work.

McConnell raised the issue of how virtual working might erode trust between team members. A lack of trust within a team might not be a serious problem in the short term, but McConnell was unsure of long-term consequences if work-from-home practices were to be mandated for six months or more.

Reinitz voiced concern for work-life balance issues, noting emergent issues with overwork and Zoom (conferencing) fatigue. Reinitz has observed team members working long hours without breaks, even though in the office they would formerly take regular breaks to play ping pong. Reinitz also observed benefits working from home – since it gives her more face time with her teenage daughter and the two often play cards during breaks. Reinitz has also observed other team members interacting with their children while in virtual meetings.

## Impact on Agile Practices

The panelists reflected on the changes in agile practices in an all-virtual work environment. McConnell observed that many agile teams are somewhat conservative and old-fashioned, using agile practices the way they were defined at the turn of the century, in the days before global distributed teams. McConnell believes that working from home and using virtual collaboration tools has “forced” teams to adopt more state-of-the-art communication practices. One area where many agile teams are progressing is in innovative uses of remote collaboration technology.

Reinitz’s team pair programmed for much of their coding work, but with the COVID-19 constraints they use a combination of approaches with some solo coding, some pairing, and some mob programming. Reinitz addressed the challenges of “mixed mode” meetings, where some attendees are face-to-face and others are remote. Her experience is that hybrid meetings require much more advance preparation. She explained that they made careful choices to select the right tools for their interactive sessions, including interactive drawing and distributed note taking. She found it beneficial to have facilitators as remote participants.

Overall, the lessons shared by the three panelists suggested that remote collaboration should be embraced. McConnell emphasized a key point from the Construx work-from-home report – “It’s really helpful to have the entire team working from home, if they’re going to work from home.” Corry agreed that all face-to-face or all virtual would be ideal, but in her experiences in Denmark, there have been more hybrid meetings. Corry warned of challenges if management creates multi-national distributed teams as a cost saving measure – merely to take advantage of differential pay scales based on geography.

## Whiteboarding and Other High-Bandwidth Collaborations

The panelists shared experiences with virtual brainstorming tools for remote collaboration. McConnell reported that many participants in the Construx work-from-home survey had mentioned the word “whiteboarding” in their text responses to the survey, so it was clear that many respondents struggled with virtual brainstorm as a replacement for face-to-face whiteboard interactions. McConnell noted that respondents characterized early design activities for conceptualization and other high-bandwidth interactions with project stakeholders as particularly challenging.

Reinitz explained that she uses multiple alternatives to traditional in-person communications including a small physical whiteboard in her home office which she uses for brainstorming and is visible via video. Participations also create drawings on paper, scan (digital photo), and then share with meeting participants. Another useful tool for shared drawings is MURAL (mural.co). Reinitz advised to be “agile” – try different approaches, leverage what works, and iterate and adjust as necessary.

Which collaboration tools are best? Simplicity and functionality are attributes often admired when assessing collaboration tools. Collaboration platforms such as Zoom, WebEx, GoToMeeting, Microsoft Teams, Skype, etc. combined with software development environments and visioning tools such as MURAL, Box Notes, Slack, and MentiMeter were important enablers for virtual work. As an aside, the XP 2020 conference applied a simple set of tools for remote collaboration: Zoom for presentations (sometimes with breakout rooms for tutorial activities), MentiMeter for quick surveys, Zoom “chat” for audience questions, and Slack for follow-up discussions.

Reinitz explained that many people find text-based communication tools such as Slack useful, but she warned that text exchanges should not be considered a replacement for face-to-face conversation. McConnell believed that people generally communicate with greater fidelity face-to-face, although introverts may communicate more readily by text with a degree of anonymity. Text interactions complemented by emojis can both avoid and cause awkward interactions.

Audience members for this virtual panel contributed to the discussion of online drawing tools and text-based communications tools. One attendee noted that drawing isn’t easy with a mouse or a touch screen. Another comment noted that groups turn to text-based collaboration tools like Slack for casual conversations in their everyday work. Slack-based dialogs are generally less effective than the conversations that co-workers would have face-to-face over lunch or in hallways, because text-based communication lacks body language cues and may be harder to interpret. It was also noted that text communication using emojis can be misinterpreted since so much depends on personal interpretation.

## Spinning up New Teams and On-Boarding New Employees

McConnell reported that the Construx survey respondents found that spinning up new teams in an all-virtual environment is difficult. New team members require high-touch interactions (a mix of coaching and mentoring) to learn and excel at their new jobs. Reinitz shared experiences for new hires at IBM’s Garage organization, explaining that their training takes more effort. Training in a remote collaboration environment requires integration of online and virtual training experiences to be effective. Virtual training is exhausting due to long hours of “screen time” – a similar challenge to that experienced by virtual conference attendees such as XP 2020 participants.

Reinitz further reflected on the training process for new employees, observing that we learn how to shape our work by watching and emulating others. In a distributed online work environment, it is necessary to be very deliberate about the act of watching others work. Reinitz believes that a technique of immersive learning for new employee onboarding can be achieved through virtual work shadowing.

## Summary

In the short term, many organizations are rediscovering Plato’s [5] observation that “necessity is the mother of invention.” The primary conclusion of the panel was that tech workers will continue to work from home and use virtual collaboration technology for the foreseeable future. High-performing teams will do well, but teams with interpersonal communication challenges will likely struggle. Many (as expressed in the popular press [6]) prefer to work in a virtual collaboration environment from their home without the need for a physical office and the overhead of commute, even if the COVID-19 crisis subsides.

Although the virtual work environment will be appealing to many knowledge workers and companies, the popular press is also beginning to warn about some of the risks and problems of a transition to a virtual environment [7, 8]. Employees are now taking personal responsible for issues usually administered by their company: e.g., office furnishings, network and compute infrastructure, workplace safety, heat, light, and power. As an aside, press reports [9] attribute world-wide shortages in toilet paper to differences in supply chains for commercial and home use.

Some companies may follow the lead of Facebook, whose CEO indicated the possibility that “employee compensation will be adjusted based on the cost of living in the locations where workers choose to live. [10]” Virtual workers are often very isolated, they have more pressure to work unpaid overtime, and it is more difficult for virtual workers to organize collectively to oppose unfair management practices. Unequal and potentially unfair compensation policies are not consistent with agile values [11]. Related issues of outsourcing and offshoring have been previously discussed at XP [12] and ACM’s OOPSLA/SPLASH [13, 14] conferences in the not so distant past.

Teams that excel in the application of agile development practices will likely succeed with the integration of virtual collaboration practices and tools into their distributed work environment. High-bandwidth interactions such as design discussions and dialogs with stakeholders will drive teams to replace standard “discussions catalyzed by a whiteboard” with new kinds of virtual interactions. Some meetings will use tools including digital cameras and physical whiteboards, while others will rely on a mix of collaborative software, digital drawing tools, and distributed annotation tools.

Meeting the challenge of building new teams and onboarding employees will require better strategies for virtual training and knowledge sharing. In many ways, COVID-19 has accelerated the adoption and deployment of network-based digital collaboration tools and new practices to ensure team and company agility – however, many of the team challenges described in Peopleware [15] and Brooks’ treatise on development practices [16] endure – and teams would be well advised to remember past lessons in the still short history of software development.
